# Novel Screw Placement Method for Extremely Small Lumbar Pedicles in Scoliosis

**DOI:** 10.3390/jcm13041115

**Published:** 2024-02-16

**Authors:** Chang-Ju Hwang, Joo-Young Lee, Dong-Ho Lee, Jae-Hwan Cho, Choon-Sung Lee, Mi-Young Lee, So-Jung Yoon

**Affiliations:** 1 Department of Orthopedic Surgery, Scoliosis Center, Asan Medical Center, University of Ulsan College of Medicine, Seoul 05505, Republic of Koreaspinecjh@amc.ac.kr (J.-H.C.);; 2 Department of Orthopedic Surgery, Dong-A Medical Center, University of Dong-A College of Medicine, Busan 49201, Republic of Korea

**Keywords:** extremely small lumbar pedicle, scoliosis, lumbar pedicle screw placement, medial margin targeting method, scoliosis surgery

## Abstract

**Study Design:** Consecutive case series. **Objective:** To propose a screw placement method in patients with extremely small lumbar pedicles (ESLPs) (<2 mm) to maintain screw density and correction power, without relying on the O-arm navigation system. **Summary of Background Data:** In scoliosis surgery, ESLPs can hinder probe passage, resulting in exclusion or substitution of the pedicle screws with a hook. Screw density affects correction power, making it necessary to maximize the number of screw placements, especially in the lumbar curve. Limited studies provide technical guidelines for screw placement in patients with ESLPs, independent of the O-arm navigation system. **Methods:** We enrolled 19 patients who underwent scoliosis correction surgery using our novel screw placement method for ESLPs. Clinical, radiological, and surgical parameters were assessed. After posterior exposure of the spine, the C-arm fluoroscope was rotated to obtain a true posterior–anterior view and both pedicles were symmetrically visualized. An imaginary pedicle outline was presumed based on the elliptical or linear shadow from the pedicle. The screw entry point was established at a 2 (or 10) o’clock position in the presumed pedicle outline. After adjusting the gear-shift convergence, both cortices of the transverse process were penetrated and the tip was advanced towards the lateral vertebral body wall, where an extrapedicular screw was placed with tricortical fixation. **Results:** Out of 90 lumbar screws in 19 patients, 33 screws were inserted using our novel method, without correction loss or complications during an average follow-up period of 28.44 months, except radiological loosening of one screw. **Conclusions:** Our new extrapedicular screw placement method into the vertebral body provides an easy, accurate, and safe alternative for scoliosis patients with ESLPs without relying on the O-arm navigation system. Surgeons must consider utilizing this method to enhance correction power in scoliosis surgery, regardless of the small size of the lumbar pedicle.

## 1. Introduction

The surgical method for scoliosis has progressed extensively, particularly in the pedicle screw placement, which plays a crucial role in surgical outcomes. Various methods, including the biplanar radiograph method [1], the freehand technique for the thoracic spine [2], the image-guided navigation method [3], and the medial margin targeting method [4], have been introduced and developed. In scoliosis surgery, placement of a sufficient number of pedicle screws is the most important factor in terms of screw density, the improvement of correction force, and the durability of correction, especially at the lumbar spine level, which is the distal aspect of the correction site. A medial margin targeting method was introduced to facilitate thoracic pedicle screw insertion in severely rotated and deformed vertebrae [4]; however, a standardized method for screw insertion in extremely small lumbar pedicles (ESLPs) is still lacking. Recently, the various imaging, navigation, and robotic technologies available for spinal fusion surgery have increased significantly. Modern assistive technology options provide high accuracy for pedicle screw placement. The introduction of the O-arm navigation system (the Medtronic O-arm II) has led to its increased utilization in patients with ESLPs, as it provides a relatively easy and safe pedicle screw insertion path for the surgeon [5]. However, the high cost of the O-arm navigation system makes it inaccessible to many tertiary hospitals. Additionally, the O-arm II, being a 3D-fluoroscopy unit, exposes patients to higher computed tomography (CT)-like radiation doses (2–2.5 mSv) [6] than that in C-arm-guided surgery. Therefore, in medical facilities without the O-arm navigation system, screw insertion in ESLPs (<2 mm) is often avoided, which causes a decrease in screw density, corrective force, and correction durability, potentially necessitating future reoperation [7].

The purpose of this study was to present a novel screw insertion method utilizing C-arm fluoroscopic guidance alone, without relying on the O-arm navigation system, and to evaluate its clinical usefulness in scoliosis patients with ESLPs.

## 2. Materials and Methods

### 2.1. Patients

After obtaining Institutional Review Board approval (IRB#2022-0999), 19 consecutive patients underwent posterior deformity correction and instrumentation using the novel screw insertion technique for ESLPs at a single tertiary hospital between 2015 and 2021 and completed a minimum 2-year follow-up. All surgeries were performed by the first author. Patients’ clinical, surgical, and radiological data were collected with attention to identify any screw-related surgical complications. Patient medical records were retrospectively reviewed to obtain demographic data and details regarding screw-related complications. Each patient underwent upright whole spine posteroanterior (PA), and lateral plain radiographies preoperatively; this assessment was repeated immediately following the surgery and at 3 months, 6 months, 1 year, and 2 years postoperatively. Additionally, CT was performed preoperatively and 3 days, 6 months, and 1 and 2 years postoperatively. At our center, routine CT was performed on the 3rd day and 6 months after the surgery to accurately determine the appropriateness of the screw position and screw loosening. CT was routinely performed for accurate judgment of operative segmental bony fusion at 1 and 2 years post surgery. All CT scans were performed with sufficient explanation to the patients, and patient consent, specifically addressing radiation exposure, was obtained prior to conducting them. All cases were monitored using somatosensory-evoked potential and motor-evoked potential during surgery.

### 2.2. Pre- and Postoperative Evaluation

All patients had their Cobb angle measured using whole-spine posteroanterior (PA) and lateral plain radiographs (prone, passive, and standing position) before and after surgery [8] and were classified according to the KING and Lenke classification methods [9,10]. Cases of syndromic scoliosis due to Marfan syndrome, etc., are listed separately. The sizes of all screws were determined preoperatively based on pedicle morphometry on CT scans. CT images were reconstructed using the Advantage Workstation (version 4.4, GE Healthcare, Wakesha, WI, USA) to visualize the center of both pedicles in one axial planar view. The narrowest transverse diameter of the pedicle between the cortical edges was measured for each thoracic and lumbar pedicle in the reconstructed axial CT image. The screw diameter was determined according to the narrowest pedicle diameter, and the screw length was determined according to the provisional trajectory. “Extremely small lumbar pedicles (ESLPs)” were defined as small lumbar pedicles < 2 mm in transverse diameter in this study. Rotation of the vertebral body was measured as the angle between the vertical line and the line bisecting the spinal canal and vertebral body on axial CT images, reconnected parallel to each spinal endplate. Screw loosening was defined as the appearance of a radiolucent rim > 1 mm around at least one screw on a radiograph or CT scan, with or without related symptoms [11]. Statistical analysis was performed using SPSS version 20.0 (SPSS Inc., Chicago, IL, USA) and MedCalc version 20.106 (MedCalc Software Ltd, Acacialaan, Ostend, Belgium). Statistical significance was set at *p* < 0.05.

### 2.3. Surgical Technique

**Figure 1:**
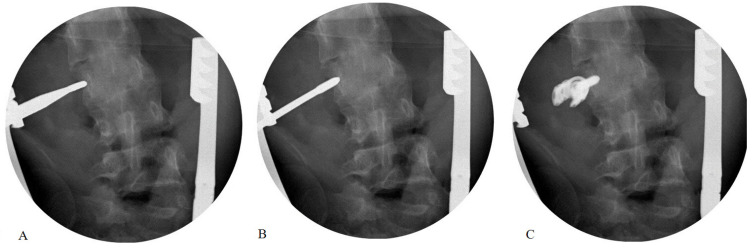
Image demonstrating the process of the extrapedicular screw placement process under C-arm guidance in extremely small lumbar pedicles (**A**–**C**).

**Figure 2:**
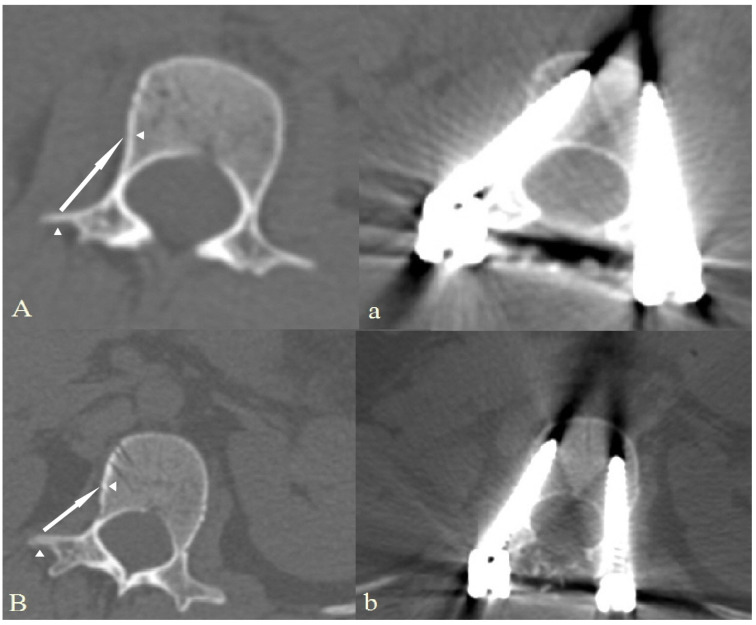
Comparative computed tomography images before (**A**,**B**) and after (**a**,**b**) screw insertion using our new technique in two patients (**A**,**B**). Each angular arrow indicates the cortical bone entry point, and long arrows indicate the trajectory of each screw.

Our technique was an advancement of the “medial margin targeting method [4]”. Following the posterior exposure of the lumbar vertebra with transverse process (TP) through appropriate soft tissue dissection, a C-arm fluoroscope was gradually rotated until a true posterior–anterior view was obtained, enabling the symmetrical visualization of both pedicles. For ESLPs, pedicle shadows appeared as long and slender ellipses or lines; an imaginary pedicle outline was presumed based on the elliptical or linear shadow. The entry point of a screw was established at a 2 (or 10) o’clock position in the presumed pedicle outline. After adjusting the appropriate convergence of gear shift, both cortices of the transverse process were penetrated and the tip was advanced towards the lateral wall of the vertebral body. If the gear-shift tip made contact with the lateral cortex, this was verified using the C-arm fluoroscope (Figure 1A). The tip should be positioned lateral to the pedicular medial margin to avoid penetrating the spinal canal. If the gear-shift tip position was appropriate, the tip was advanced to an additional 15–20 mm through the lateral cortex (Figure 1B). After creating an internal entry point in the lateral cortex body, all bony borders were checked by palpating them before and after tapping with a probe. Subsequently, an extrapedicular screw was placed into the vertebral body using tricortical fixation (Figure 1C and Figure 2). If the TP and pedicle level did not match or there was no TP due to deformity, the gear shift was adjusted to the pedicle level with an appropriate convergence angle and advanced to make contact with the lateral cortex of the body at an insertion depth of approximately 20 mm. This is because the gear-shifting length is usually 20 mm when using the free-hand technique for pedicle screw insertion [2]. Following that, lateral cortical body fixation was performed, as previously mentioned.

## 3. Results

**Figure 3:**
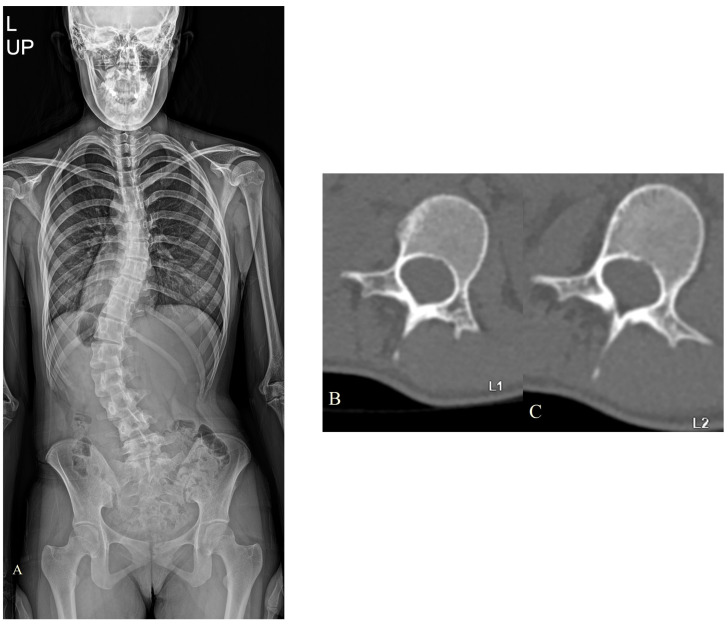

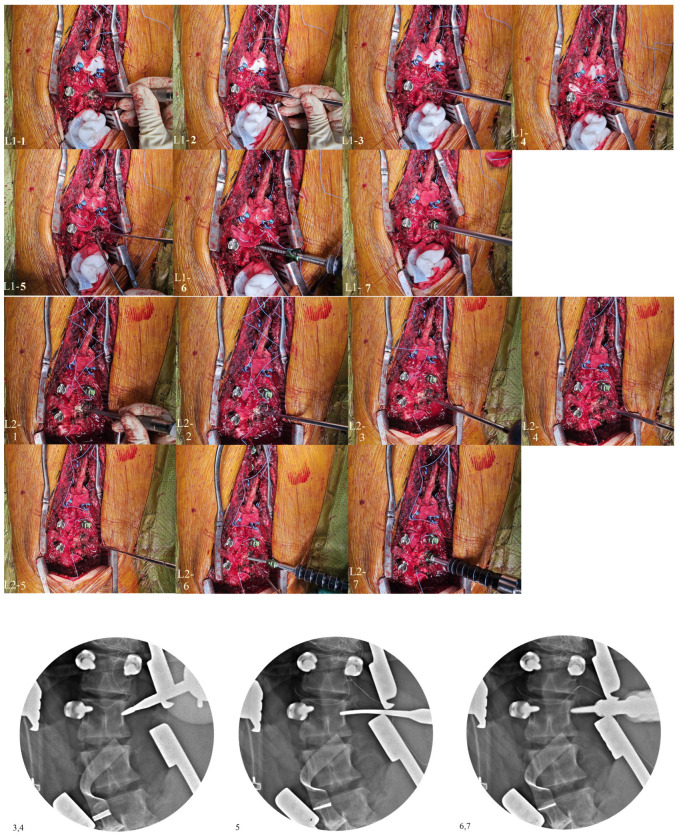

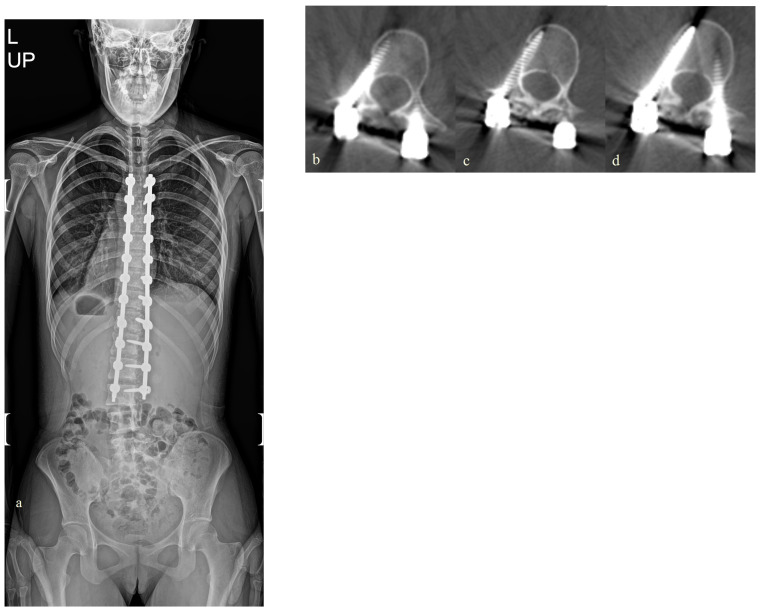
Images demonstrating a 15-year-old female AIS patient’s operative case. Preoperative images: upright, whole-spine PA radiographs (**A**), L1 axial cut CT image demonstrating ESLP, Rt. (**B**), and L2 axial cut CT image demonstrating ESLP, Rt. (**C**). Intraoperative images (upper line—L1, middle line—L2, and bottom line—C arm image): 1. Image of creating a shallow entry using a burr on the exposed TP to prevent the gear shift from slipping. 2. Image of penetration after positioning the gear shift at the entry point on the TP. 3. Image of the gear shift completely penetrating the TP and touching the cortical bone outside the vertebral body. 4. Image showing the gear shift inserted 15–20 mm further into the vertebral body. 5. Image of palpation of all bony borders using the probe. 6. Image where the screw is positioned at appropriate convergence in the corresponding entry. 7. Image showing fully inserted screws. Post-operative images: upright, whole-spine PA radiographs (**a**), L1 axial cut CT image demonstrating screw placement using this method (**b**), and L2 axial cut CT image demonstrating screw placement using this method (**c**,**d**).

**Figure 4:**
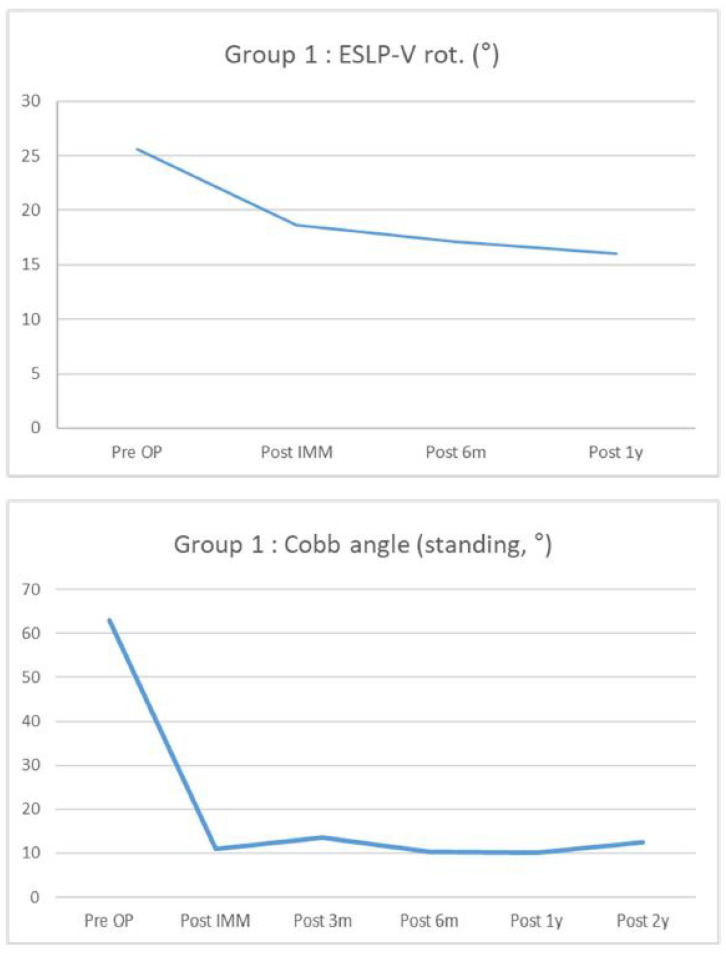
A graph showing the rotation rate of the vertebra with extremely small lumbar pedicles measured using computed tomography from the preoperative period to the 1-year postoperative period (**top**), and a graph showing changes in Cobb angle from the preoperative period to the 2-year postoperative period (**bottom**) in Group 1.

**Figure 5:**
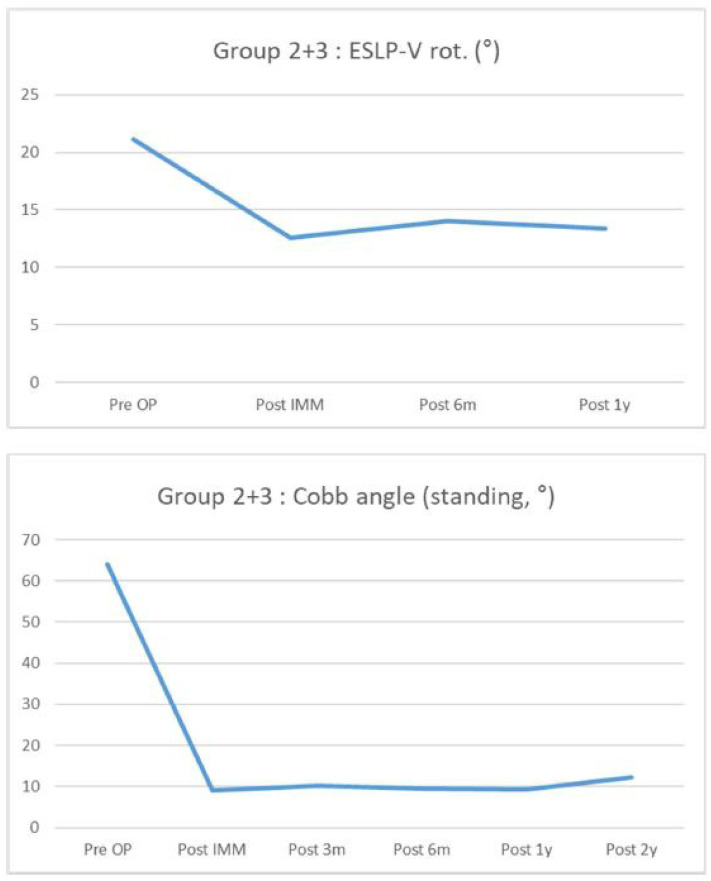
A graph showing the rotation rate of the vertebra with extremely small lumbar pedicles measured using computed tomography from the preoperative period to the 1-year postoperative period (**top**), and a graph showing changes in Cobb angle from the preoperative period to the 2-year postoperative period (**bottom**) in Group 2 + 3.

**Figure 6:**
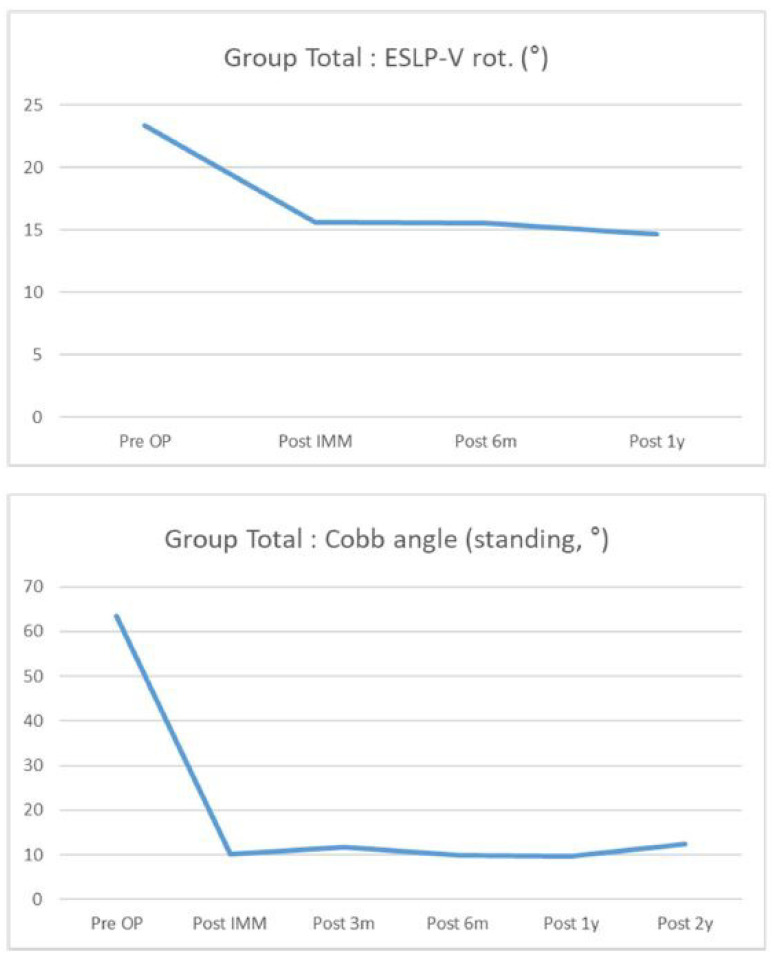
A graph showing the rotation rate of the vertebra with extremely small lumbar pedicles measured using computed tomography from the preoperative period to the 1-year postoperative period (**top**), and a graph showing changes in Cobb angle from the preoperative period to the 2-year postoperative period (**bottom**) in all patients.

ESLPs were retrospectively reviewed in 12 female and 7 male patients who underwent surgery for screw insertion with the novel method. Among the 19 patients, adolescent idiopathic scoliosis (AIS) was diagnosed in 10 patients, confirmed Marfan syndrome was diagnosed in 6 patients, suspected Marfan syndrome was diagnosed in 1 patient, and congenital kyphosis was diagnosed in 1 patient. Of the 90 lumbar screws inserted, 33 screws were inserted using the new method. The average follow-up period for all patients was 28.44 months. The most frequent lumbar level with ESLP was L1 (11/27, 40.7%), and, on average, it was rotated approximately 22.6°. Preoperatively, the average Cobb angle, while standing, was 63.5°, and the distribution of these values is shown in Table 1. Table 2 shows the distribution of fusion levels performed during surgery, posterior column osteotomy levels, and the number of screws inserted per level. In the case of only one ESLP, the same size screw as the screw on the opposite side was used in all cases, whereas if ESLPs were present on both sides, the intact bony wall was checked with the probe after gear-shifting, and the length was directly measured to determine the suitable screw size. Based on the total number of screws inserted into the ESLP-V (vertebra of extremely small lumbar pedicles), the patients were divided into Group 1 (*n* = 9) and Group 2 + 3 (*n* = 10). The degree of correction and maintenance of the Cobb angle, measured using whole-spine standing PA radiography, was investigated preoperatively and approximately 2 years postoperatively. The degree of correction and maintenance of the rotation angle of the ESLP-V, measured using CT, was investigated preoperatively and approximately 1 year postoperatively. Both Group 1 and Group 2 + 3 demonstrated excellent correction rates and maintenance of correction postoperatively (Figure 3 and Figure 4), and the same results were observed in the group total (*n* = 19) (Figure 5 and Figure 6). However, 1 year following surgery, the L2 screw of one patient (patient Q) revealed radiographic screw loosening on CT, although no loss of correction or clinical symptoms were observed at the 2-year follow-up assessment (Table 2).

## 4. Discussion

Over the past 40 years, the use of pedicle screws for scoliosis surgery has become a popular fixation method. Currently, pedicle screws are safe and effective for the posterior correction of scoliosis, providing superior spinal deformity correction in the coronal, sagittal, and axial planes, compared to that of hook and hybrid instruments, and maintaining correction parameters during follow-ups [12,13]. Lehman et al. conducted studies on cadaveric thoracic vertebrae and demonstrated that pedicle screw fixation provides strong pull-out strength to the spine [14,15]. In addition, Kuklo et al. observed no significant correlation between an increased incidence of major neurological complications and pedicle screw insertion [16]. Therefore, ensuring the correct placement of the maximum number of pedicle screws is crucial because a high screw density can provide an increased correction force [17].

However, not all pedicles can be easily inserted with pedicle screws. In 1993, Dvorak et al. introduced and developed the extrapedicular thoracic pedicle screw fixation technique [18], and later Lee et al., reported that approximately 7.8% of the total thoracic pedicles encountered during scoliosis surgery were extremely small pedicles measuring <2 mm. In addition, in the present case, an innovative method of pedicle screw insertion under C-arm guidance using the “medial margin targeting method” was presented [4]. Although, several studies have reported on screw insertion methods for extremely small pedicles in the thoracic spine, but only few studies have reported on the incidence or screw insertion methods for extremely small pedicles in the lumbar spine.

The thoracic and lumbar vertebrae have a profound structural difference, besides the difference in basic anatomical structure, which is the presence of ribs. This pedicle–rib complex provides several advantages in the pedicle screw insertion method [19]. For instance, in the thoracic spine, the “in-out-in” technique is often utilized, where screws are intentionally placed more laterally to decrease the risk of medial breach and potentially increase bony rib purchase [20]. However, in the absence of a pedicle–rib complex in the lumbar spine, no additional structure can be used to increase bone purchase. Therefore, if extremely small pedicles are encountered in the lumbar spine during scoliosis surgery, the options are either to abandon screw insertion into the pedicle or attempt pedicle screw insertion using lamina hooks, the sublaminar wiring technique, or the O-arm navigation system. However, owing to the increased cost of the O-arm navigation system, they are not available in many tertiary hospitals [6]. Considering the importance of the lumbar spine in scoliosis surgery, ensuring a maximum number of screw insertions is crucial to achieve sufficient corrective force. In this context, the novel lumbar screw fixation method proposed in this study demonstrates sufficient fixation force, maintenance, and corrective power, although not to the same extent as that of conventional pedicle screws. Due to the structural characteristics of an extremely thin pedicle, achieving a fixation force comparable to that of a pedicle screw inserted into a pedicle of normal thickness is challenging, irrespective of the method used. Screws inserted into the ESLP-V using only C-arm guidance, available at any hospital, demonstrated excellent corrective force and maintenance without loosening, with the exception of one case of radiographic screw loosening during the 2-year follow-up period. Even in this case, screw loosening was confirmed on a CT scan and did not lead to screw pull-out or clinical symptoms. Considering these results, the novel screw insertion method proposed in this study provides sufficient clinical value.

Expected disadvantages or complications include the possibility of irritation of the psoas muscle along the screw trajectory and the risk of screw pull-out due to limited bony purchases. In terms of psoas muscle irritation, no patient in our study experienced symptoms, such as a decrease in hip flexor muscle grade, pain, or lumbar plexus irritation postoperatively. This can be attributed to the slower screw insertion process and proper adjustment of the gear-shift tip direction, which minimizes the possibility of muscle damage. Additionally, the possibility of segmental artery injury exists, but no cases have been reported yet. However, the possibility of segmental artery injury should always be considered during the screw insertion process along with careful monitoring for bleeding. No case of screw pull-out was observed, which could be attributed to the high bone density in the young patient group. Therefore, it can be concluded that the fixation force for correcting and maintaining the correction of scoliosis can be obtained by utilizing fixation involving only the TP and vertebral body.

The limitations of this study include the small sample size of the study, as data were collected from a single center and included cases from a single surgery, and a relatively short follow-up period, considering the characteristics of scoliosis patients who underwent surgery at a young age. In addition, since the fixation force of the screw inserted using the new method has not been confirmed through in vitro testing, accurate quantification of the actual fixation force when employing this method is crucial.

In conclusion, the challenge of screw insertion in ESLPs, during scoliosis surgery, has posed several limitations in the past. A possible solution was obtained with the advent of the O-arm navigation system. However, this medical equipment is not easily available in many hospitals, and many spinal surgeons still require an effective screw insertion method using only C-arm guidance. The proposed method in this study may offer a unique and invaluable option for spinal surgeons attempting to insert screws into ESLPs using only C-arm guidance during scoliosis surgery.

## Figures and Tables

**Table 1:** Preoperative demographics and radiological parameters.

	Age/Sex	Diagnosis	ESLP-V Level	ESLP Rot. (°)	Cobb Angle (Standing, °)	Cobb Angle (Prone, °)	Cobb Angle (Passive, °)	King Type	Lenke Type
A	14/F	AIS	L2	45	70	45	54	5	6C
B	22/F	AIS	L1	13	71	60	61	5	4B
C	13/M	AIS	L1	32	60	45	51	1	5C
D	16/M	AIS	L1	28	55	31	26	1	5C
E	15/F	AIS	L1	22	60	40	50	1	6C
F	20/F	AIS	L1	15	62	44	49	1	3C
G	13/F	AIS	L1	23	55	35	41	1	5B
H	15/M	AIS	L3	19	65	38	51	1	5B
I	19/M	AIS	L2	33	69	40	48	5	6C
J	14/F	Marfan scoliosis	L1	33	66	45	25		
			L2	17					
K	15/F	Marfan scoliosis	L2	15	60	41	32		
			L3	19					
L	10/F	Marfan scoliosis	L1	28	71	50	41		
			L2	22					
M	17/M	AIS	L1	33	62	43	49	1	5C
			L2	25					
N	15/F	r/o Marfan scoliosis	L2	19	50	30	19		5C
			L3	22					
O	14/F	Marfan scoliosis	L1	34	66	45	58		
			L2	28					
P	10/F	Marfan scoliosis	L1	16	93	59	59		
			L2	15					
Q	13/F	Marfan scoliosis	L2	24	71	40	51		
			L3	18					
R	13/M	Congenital kyphoscoliosis	L2	4	37	34	35		
			L3	8	kyphosis 72		kyphosis 65		

AIS, adolescent idiopathic scoliosis; ESLP, extremely small lumbar pedicles; V, vertebra; Rot., rotation.

**Table 2:** Surgical procedure and screws inserted into the ESLP-V.

	Fusion Level	PCO Level	Screws Inserted into the ESLP-V	Screw Size (mm)
1 screw				
A	T3-L4	T12-L3	L2, 1ea	5.5 × 35
B	T2-L1	T6-11	L1, 1ea	5.5 × 45
C	T3-L4	T12-L3	L1, 1ea	5.5 × 45
D	T5-L4	T12-L4	L1, 1ea	5.5 × 45
E	T3-L1	T6-10	L1, 1ea	5.5 × 45
F	T2-L2	T9-L1	L1, 1ea	5.5 × 45
G	T3-L3	T10-L2	L1, 1ea	5.5 × 40
H	T3-L4	T12-L3	L3, 1ea	5.5 × 40
I	T5-L4	T11-L2	L2, 1ea	5.5 × 35
2 screws				
J	T2-L3	T8-L2	L1, 1ea	5.5 × 40
			L2, 1ea	5.5 × 40
K	T10-L4	T11-L3	L2, 1ea	5.5 × 40
			L3, 1ea	5.5 × 35
L	T2-L4	T9-L2	L1, 1ea	5.5 × 35
			L2, 1ea	5.5 × 45
3 screws				
M	T3-L3	T11-L3	L1, 1ea	5.5 × 35
			L2, 2ea	5.5 × 40
N	T10-L4	T12-L3	L2, 2ea	5.5 × 40
			L3, 1ea	5.5 × 40
O	T2-L3	T10-L2	L1, 2ea	5.5 × 40
			L2, 1ea	5.5 × 40
P	T2-L4	T9-10	L1, 1ea	5.5 × 35
			L2, 2ea	5.5 × 35
Q	T2-L4	T9-L3	L2, 1ea	5.5 × 35
			L3, 2ea	5.5 × 35
R	T9-L4	L2	L2, 2ea	5.5 × 35
			L3, 1ea	5.5 × 40

PCO, posterior column osteotomy; ESLP, extremely small lumbar pedicles; V, vertebra; screw size, diameter × length (mm); red-colored screw: radiological loosening at 1 year postoperatively on computed tomography.

## Data Availability

The datasets generated and/or analyzed during the current study are not publicly available, but are available from the corresponding author upon reasonable request.
